# A fast and efficient gene-network reconstruction method from multiple over-expression experiments

**DOI:** 10.1186/1471-2105-10-253

**Published:** 2009-08-17

**Authors:** Dejan Stokić, Rudolf Hanel, Stefan Thurner

**Affiliations:** 1 Complex Systems Research Group, Medical University of Vienna, Währinger Gürtel 18–20, A-1090, Austria; 2 Santa Fe Institute, 1399 Hyde Park Road, Santa Fe, NM 87501, USA

## Abstract

**Background:**

Reverse engineering of gene regulatory networks presents one of the big challenges in systems biology. Gene regulatory networks are usually inferred from a set of single-gene over-expressions and/or knockout experiments. Functional relationships between genes are retrieved either from the steady state gene expressions or from respective time series.

**Results:**

We present a novel algorithm for gene network reconstruction on the basis of steady-state gene-chip data from over-expression experiments. The algorithm is based on a straight forward solution of a linear gene-dynamics equation, where experimental data is fed in as a first predictor for the solution. We compare the algorithm's performance with the NIR algorithm, both on the well known *E. coli *experimental data and on in-silico experiments.

**Conclusion:**

We show superiority of the proposed algorithm in the number of correctly reconstructed links and discuss computational time and robustness. The proposed algorithm is not limited by combinatorial explosion problems and can be used in principle for large networks.

## Background

Prediction of functional relationships between genes, starting from actual gene expression data, is one of the primary goals of systems biology. Despite large efforts in this direction [1,2], either based on transcription factor – promoter interaction [3,4], or on inferring gene networks [5-9], methods for reliable predictions of collective behavior of gene-activity are yet to be found. Some general facts about the topology of gene regulatory networks [10-12], statistics of gene expressions [13] or the dynamics of gene regulation [5] are becoming to be understood. This knowledge is far from sufficient to successfully reconstruct gene networks, but can be helpful in limiting the tremendous number of parameters involved in reconstruction. Even if the average degree of the gene regulatory network, i.e. the number of genes regulated by some gene on average, was known, noisy and limited data will always lead to severe problems. The degree *k*_*i *_of a node *i *in a network is defined as the number of links that emerge from -or point to- that node. The average degree is denoted by ⟨*k*⟩.

There are basically two types of reverse engineering approaches depending on the experimental setup, inferring the gene network from steady-state [8,9] or from time-series [14,15] experiments. By using steady-state experiments, one can not draw any conclusion about the dynamics of gene regulation. Conducting time-series experiments gives helpful insights into gene regulatory dynamics, but often with the price of getting redundant information. Further, due to costs full time-series data on gene expression are in general not available. As described in [1], one can further divide the reverse engineering methods into four categories: differential equation models [5,6], boolean network models [7], Bayesian network models [8] and association networks [16]. The reverse engineering methods based on differential equations further may rely on linear [9] or nonlinear differential equations [17]. The latter models may possess potential in alleviating the effects of insufficient information fundamental in reverse engineering of genetic networks by obtaining information from combinatorial perturbations measurements on the system. In linear systems such information would be redundant.

How can gene regulatory network reconstruction methods be validated and compared? Neither a standardized biological benchmark, nor a consensus on what class of models to use for in-silico testing exists [1]. The usual way is to validate a method either by applying it on a given experimental dataset or on in-silico datasets. In both cases one has to deal with different problems. Applying a method to an experimental dataset, poses the problem of comparing the reconstruction result with a network which is always just a consensus on how a biological network *could *look like, but never the exact gene regulatory network. On the other hand, when applying a reconstruction models to in-silico data, one has a perfect reference network, however the generated timeseries data is a result of a dynamical model of gene interaction, which cannot be shown to overlap with the real gene regulation dynamics.

In this paper we introduce a novel reverse engineering algorithm and validate it against biological and in silico data. The performance of the algorithm is compared against *Network identification by multiple Regression *(NIR) [9] for several reasons. i) NIR is considered a state of the art algorithm which plays a role comparable to a benchmark. To our best knowledge NIR has not been outperformed by other algorithms with respect to predicting the topology of genetic networks (real and in-silico) correctly -on average- so far. However, NIR has two drawbacks. a) The computation time increases quickly with the network size. b) NIR can not identify hubs in networks. We will briefly discuss this in a little more detail further below. Recently a reconstruction algorithms has been proposed by [18] which is clearly faster than NIR. Yet, its predictions do not better or completely equal the ones made by NIR. Another algorithm has been proposed recently [8], which correctly identifies the recA-hub in the E. coli SOS response network and reports a higher statistical significance of the reconstructed SOS network. Yet, the algorithm is restricted to a-cyclic networks. Both cases lack a comprehensive comparison with NIR and do not challenge its role as benchmark. ii) We decided to selected an *E. coli *dataset [9] for biological validation of our algorithm. This choice is satisfactory since the underlying SOS response network has been subject to over 30 years of research, which provides us with a well established consensus about the actual gene regulations going on in that particular sub-network. Moreover NIR has been applied to the same SOS response network of *E. coli *which makes it possible to compare the two algorithms. iii) Both, the proposed algorithm and NIR, build on the assumption that the dynamics of RNA concentrations defined by the underlying network is governed by a linear differential equation. For generating in silico data we therefore are using a linear gene regulation model proposed by the authors [19]. The only nonlinearity in the model is introduced by the condition that RNA concentrations can not become negative. This model simultaneously captures a series of experimental facts, such as the distribution of genome wide gene-expression levels, multi-stability and periodicity. The proposed reconstruction algorithm does not rely on the detailed knowledge of the data generation model. The generated in silico data therefore is realistic and allows for an unbiased comparison of the proposed algorithm with NIR.

Although the proposed algorithm and NIR start from identical assumptions on RNA concentration dynamics and equally have to deal with insufficient information the reconstruction approaches are in fact quite different. The inference idea of the NIR algorithm is to reconstruct the network by using a least-squares multiple regression approach assuming the same fixed number of regulatory links for every gene, i.e. the average degree ⟨*k*⟩ of the network. Basically this means that promising ensembles of links are put to the test and the ensemble which gives the least squared error with the input data on basis of the underlying linear model is selected as the optimal solution which is calculated for all the () possible combinations, where *N *is the size of the network. This combinatorial factor is responsible for a polynomial time characteristics of NIR, i.e. roughly *T *∝ *N*^⟨*k*⟩^, which limits its applicability to relatively small networks with low average degree. Gene-networks are expected to have average degrees of about 4. For average degree ⟨*k*⟩ = 4 this implies that computation time of NIR grows quickly with at least a factor *N*^4^. Moreover, since NIR only considers networks where all nodes have identical degree there is no way NIR could detect hubs in networks. Clearly NIR will perform best on networks with sharply peaked degree distributions and perform bad on networks with broad degree distributions. The proposed algorithm however relies on the observation that for very sparse networks with no loops the information provided by experiments (perturbations and associated RNA concentrations) is sufficient to infer the network. The proposed approach tries to extrapolate the sparse network predictions to non sparse networks guided by the exact solutions of the linear differential equations. In contrast to NIR the number of regulatory links per gene are not fixed a priory which intuitively is much more realistic. While large networks of even thousands of genes do not actually pose a problem for the proposed algorithm in terms of computing time there are of course limitations to the network size in terms of available independent experimental information. Basically () link weights have to be estimated from maximal *N *independent over expression experiments. Any reverse engineering algorithm relying on this limited information will necessarily approach the results of pure gambling with growing network size.

## Methods

A system of interacting genes can be seen as a complex network, where every directed link represents a functional relationship between two genes. For simplicity, let us assume that this link will contain both transcriptional and translation levels of gene interactions. In this oversimplified view one can assume that the gene expression level changes in time as

where *g*(*X*) is an a priori unknown function of a time dependent vector of gene expression levels *X*. If there are *N *genes in the (sub)network under study (e.g. *N *genes on a custom chip), vector *X *has *N *components. If we assume, as in [14], that *g*(*X*(*t*)) is a linear function (or after linearization of a more complicated function) one can write Equation (1) as

where *μ *is a vector of gene over-expressions and *A *is a constant adjacency matrix, containing the "strength" of gene-gene interaction. The elements *A*_*ij *_can be positive or negative real numbers, indicating activating or inhibiting interactions, respectively. By solving Equation (2) one formally gets

Where superscript *μ *indicates the system was perturbed with the constant vector *μ*. After *M *over-expressions, one can write the above equation in matrix form

where  is the collection of all gene expression levels after the *M *over-expressions experiments, organized in a *N *× *M *matrix, where one of the *M *columns is a time dependent *N*-vector of gene expression levels for different gene being over-expressed. In the following let us assume that we are able to perform *N *over-expression experiments, i.e. *M *= *N*.  is a *N *× *N *diagonal matrix of gene over-expression levels.  is diagonal, because in every over-expression experiment just one gene is being over-expressed (which is the experimentally feasible case). At this point we emphasize that even though we know from the way over-expression experiments are prepared that the matrix  is diagonal, one often has little to no experimental control about the exact amplitude of its entries. This problem is mitigated for small times *t *≪ 1. To see this we define

Using this definition and abbreviating  ≡ *At *Equation (4) can be rewritten into *Q *= *A*^-1^(*e*^*At *^- *I*)/*t *and consequently into

It is easy to check that in the short time limit

holds. For very short times t our lack of knowledge is thus basically irrelevant and estimating *Q *reduces to estimating . Yet, for 1 ≫ *t *we have 1 ≫ || and Equation (6) effectively reduces to the trivial identity  = , i.e. for short times Equation (6) is consistently fulfilled but provides no constraining information on . However, when the matrix is so sparse that each node has at most only one regulatory link and no loops are present in the network the relative responses

will be one-to-one related with the network topology.  is the gene expression level of the *i*th gene when no perturbation has occurred (*μ *≡ 0) and  the gene expression level of the *i*th gene, where the *j*th gene has been over-expressed. It is also not hard to realize that for sparse adjacency matrices  the relative response will in general provide a good first estimate, i.e.  ∝ *Y*. Moreover, linearity assures that relative responses for short times will be small, |*Y*_*ij*_| ≪ 1 and therefore *Q *~  ~ *Y*.

However, for times in the order of a cell-cycle *t *~1 and less sparse matrices these estimates will not be sufficient, i.e. the *Y*_*ij *_will not in general be small and *Q *need not be close to *I*. The idea is to replace *Y*_*ij *_by some function *f*(*Y*_*ij*_) and estimate *Q *with *D*_*ij *_≡ *f *(*Y*_*ij*_). The function *f *clearly should have the properties that (i) *f*(*Y*_*ij*_) ~*Y*_*ij *_for |*Y*_*ij*_| ≪ 1 and (ii) *f *is a monotonously increasing function. Since in practice *Y*_*ij *_can range over many decades in amplitude we also presume that (iii) *f *should be a concave function. Lastly, (iv) *f *has to be defined on [-1, ∞] since -1 ≤ *Y*_*ij*_, but in principle could be arbitrary large for positive values and map [-1, ∞] to [-∞, ∞]. Maybe the simplest function fulfilling this requirements is the logarithm, i.e. *f*(*x*) ≡ ln(1 + *x*), and *D *gets

This means that we effectively estimate  = ln(*I *+ *D*), where I is the identity matrix. For the matrix logarithm to provide unique solutions, *I *+ *D *should not have any negative real eigenvalues. Since experimental results show that this is not the case in general we use a *cleaned *version (see below) of *D*, denoted by *D*^0 ^such that *I *+ *D*^0 ^has no negative real eigenvalues and the prediction of the adjacency matrix is given by

### Eigenvalue cleaning

In general, the logarithm of a matrix can have an infinite number of real and complex solutions. In order to find a unique solution of ln(*D *+ *I*), matrix *D *+ *I *can not have negative real eigenvalues. If we take a look at the eigenspectrum of matrix *D *from various experiments, both biological and in-silico, we notice that most of the eigenvalues are complex, however a small number of eigenvalues are real, both positive and negative. Thus, we first have to clean the matrix *D *+ *I*, meaning to set all the negative eigenvalues to small positive number *ϵ*. This is done by first diagonalizing matrix *D*:

where the eigenvalues are ordered in a way that first *L *eigenvalues are real and less then -1,  = *d*_*i *_< -1, ∀*i *= *L*. These *L *diagonal elements are set to *ϵ *- 1

and are rotated to yield the cleaned matrix

Matrix *D*^0 ^+ *I *no longer has negative real eigenvalues, and a unique prediction of an adjacency matrix *A*-reconstructed gene regulatory network – can be given

### Thresholding

Our solution *A *will in general represent a fully connected network, with a certain distribution of link weights around zero. The reason why we are always getting fully connected network, e.g. network without zero entries in adjacency matrix, is because of the noisy measurements. Real gene regulatory networks are never fully connected, but are characterized by an average degree ⟨*k*⟩, which has been estimated to be relatively small ~2 – 4 [12]. For simplicity we assume ⟨*k*⟩ for the undirected unweighted case. Knowledge of ⟨*k*⟩ allows to define a clear thresholding scheme. All entries in *A *below a threshold *α *are set to zero. *α *is chosen such, that matrix  has the average degree ⟨*k*⟩, i.e.

such that

*A*^0 ^is the first approximation of the gene regulatory network we want to reconstruct.

### A note on fewer than *N *experiments

In the case where the number of over-expression experiments *M *is lower than the size of the network *N*, matrix *D *is not quadratic, thus we are unable to calculate the matrix logarithm. Information about the influence of gene *j *(*j *> *M*) on gene *i *is missing. A way around is that one can introduce a measure of the distance between two genes in the network. Although the correlation between gene expressions in different over-expression measurements can not lead to any conclusion about the functional relations among genes, it can provide a good measure for the distance between the genes in the network, where strong correlations means low functional distance. One can therefore simply calculate a matrix of correlation coefficients and replace the missing terms in *D*:

Here the first index in *D*_*ij*_, *i *runs in the domain *M *<*i *≤ *N*, the second index, 1 ≤ *j *≤ *N*.

### Testing the method

We have run both, the proposed algorithm and NIR on a usual PC (AMD Athlon XP 3000+, 1 GB RAM, SUSE-LINUX 9.0, KDE 3.1 environment) using MATLAB 6.0. We compare our results with the NIR algorithm both on an *in-silico *dataset, as well as on the *E. coli *SOS response network [20-24], in the same way as in [9]. We measure performance in two ways, firstly, by counting the fraction of correctly reconstructed positive, negative and zero links, denoted by F_+_, F_- _and F_0_, respectively. For later use we define F ≡ F_+ _+ F_- _+ F_0_. Secondly, by calculating the extended Matthews correlation coefficient [25], a discrete version of Pearson's correlation coefficient, extrapolated onto *K *× *K *confusion matrices. Matthews correlation coefficient is taking values in the interval [-1, 1], where 0 stands for no correlation between predicted and real case, and 1 and -1 stands for complete or negative correlation respectively. The *K*-category correlation coefficient is defined as

where *C *is a *K *× *K *confusion matrix, or more precisely the element *C*_*kl *_is counting the number of cases where category *k *is predicted, but category *l *was present. In our case, *K *= 3 and the categories are: positive link, negative link and no link between any two genes. It is straight forward to see that p-values for any value of *R*^*K *^can be computed exactly in the same way as for the Pearson correlation coefficient, provided sample size is given.

It is important to stress the difference in measuring reconstruction performance in *in-silico *and biological experiments. While in biological networks, self-regulation is a part of the complete gene regulatory network, in numerically simulated gene regulation dynamics, self-regulation is often screened by negative self-degradation rates, which have to be imposed, in order to keep the dynamics sufficiently stable, see e.g. [19]. To be as correct and conservative as possible, we therefore compare our reconstructed adjacency matrix *only *with the off-diagonal elements in the *in-silico *case. In the *E. coli *case we of course compare with the complete adjacency matrix.

#### In-silico testing

**Figure 1:**
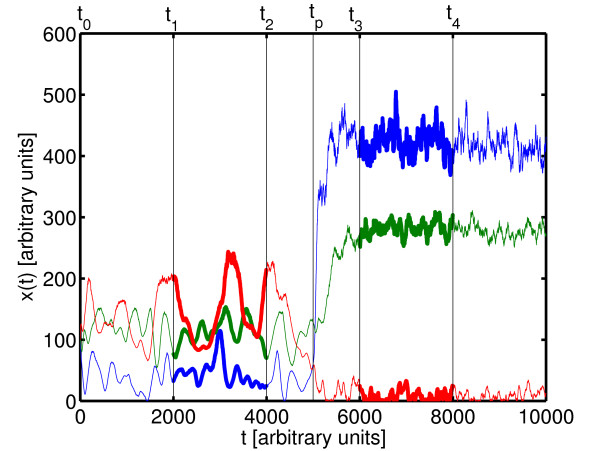
**Time series of model-gene activity**. Time series of three randomly selected trajectories (numerical solutions of Eq. (19)), showing the measurements of gene expression levels in *in-silico *over-expression experiment. Gene expression levels were measured from time *t*_1 _until *t*_2 _for the steady state levels, and from time *t*_3 _until *t*_4 _for the effect of perturbation. At the time *t*_*p *_one gene was over-expressed.

We employ a recently proposed dynamical gene-gene interaction model, which is able to capture a series of experimental facts on gene-expression statistics [19]: (i) distribution of gene-expression increments over time, (ii) multiple equilibria, (iii) stability. The model is defined as

with a positivity condition imposed for gene expression levels (non negativity of concentrations):

Here, *A*^*model *^is a real valued adjacency matrix of gene-gene interactions. It is modeled as a particular random matrix, mimicking experimentally known facts [19]. *x*(*t*) is a vector of gene-expression levels in time *t*, constant vector *x*^0 ^indicates steady state gene-expression levels. *ξ *and *η *are multiplicative and additive noise terms, respectively, which are a generic feature in chemical reactions. Using the dynamics defined in Equation (19) we generate the time series of gene expression levels *x*(*t*), and simulate the effects of perturbation by adding a constant perturbation vector to the Equation (19). For details, see [19]. We *measure *the gene expression levels as time averages over concentrations:  and , where *t*_0 _<*t*_1 _<*t*_2 _<*t*_*p *_<*t*_3 _<*t*_4_. *t*_0 _is the initial time point of the simulation (after discounting transient behavior), *t*_*p *_is the time at which the perturbation vector (with the *j*th component being non zero) is applied. The procedure is depicted in Figure 1.

#### Testing on the *E. coli *dataset

We use the wild-type *E. coli *strain MG1655 available at [9]. The reason for testing our method on this particular dataset is the fact that the SOS response of the *E. coli *is well understood, and some consensus over the topology of its gene regulatory network is reached. Moreover it is possible to compare reconstruction success with other groups [8,18,26]. We test the performance by counting the fraction of the correctly reconstructed links of all three classes (positive, negative and zero), and with the extended Matthews correlation coefficient.

#### The pure-chance reconstruction threshold

A strong criterion of checking the performance of any reconstruction method we consider, is to compare it with a pure random-reconstruction. Several proposed gene network reconstruction algorithms can be shown to perform only slightly above pure-chance reconstruction. Random reconstruction can be performed in the following way. Suppose that ⟨*k*⟩ denotes the true average degree of the network, which may or may not be known, and *k*_*g *_denotes a guess on ⟨*k*⟩. Since we estimate that the directed network has *L *= *Nk*_*g *_links we take a fully connected network and assign a random order to all *N*(*N *- 1) links. Then we take a random number with three outcomes: + (positive weight), - (negative weight), and 0 (no link), and assume that there are as many positive as negative links. The distribution of these outcomes therefore is such that both + and - occur with probability *w*_± _= *k*_*g*_/2*N*, while the 0 appears with probability *w*_0 _= 1 - *k*_*g*_/*N*. The *true *probabilities, i.e. the probability of +, -, 0 if the true average ⟨*k*⟩ was known, however are, *p*_± _= ⟨*k*⟩/2*N *and *p*_0 _= 1 - ⟨*k*⟩/*N*. Now we pick one link after another in the given random order, and assign a random symbol, +, - or 0 and repeat this until *L *links have been assigned either + or -. Since 'throwing the dice' is an event independent of the network topology, one can simply compute (*k*_*g*_|⟨*k*⟩) = *w*_± _*p*_± _and (*k*|⟨*k*⟩) = *w*_0_*p*_0_.

If reconstruction is based on pure chance the expected *K*-category correlation will be *R*^*K *^= 0. This can be seen by inserting the confusion matrix *C*_*ij *_= *w*_*i*_*p*_*j*_, *i *and *j *indexing +, - or 0, into Equation (18).

## Results

### Reconstruction on in-silico data

**Figure 2:**
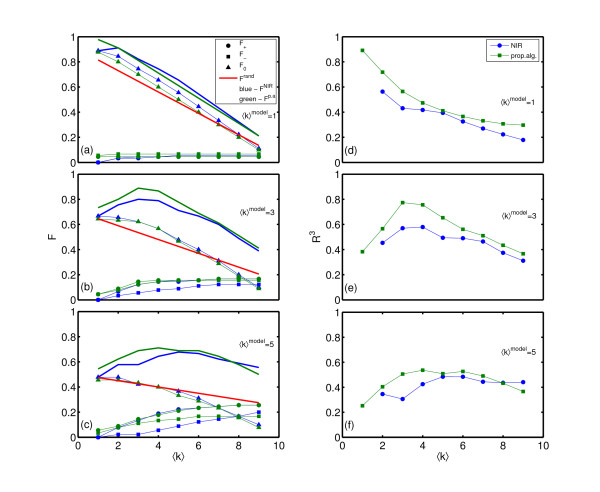
**Network reconstruction and comparison: in silico**. Results of network reconstruction for the proposed algorithm (green lines) and NIR (blue lines) for in-silico experiments. Results for the fraction of correctly reconstructed links ((a)-(c)), and extended Matthews correlation coefficient ((d)-(f)) are shown. Three in-silico networks with different average degree were constructed, for ⟨⟩ equals 1((a),(b)), 3((b),(e)) and 5((c),(f)). In the plots where the fraction of correctly reconstructed links are shown, circles denote the fraction of positive links *F*_+ _, squares the fraction of negative links *F*_- _and triangles no links *F*_0_. The red line represents the gambling threshold *F*^*rand*^.

We generated networks (*N *= 10) with three different connectivities (⟨⟩ ∈ {1, 3, 5}), for purposes of in-silico testing of our reconstruction algorithm. Using the generated adjacency matrices *A*^*model *^of these networks we, simulated time series of gene expression levels (see Figure 1) according to Equation (19), with noise levels *σ *=  = 0.1, where *ξ*_*i *_∈ *N *(0, ) and *η*_*i *_∈ *N *(0, *σ*). For details, see [19]. As described in the previous section, we measured the steady state gene expression levels before and after the perturbation of each gene in the network, denoted by  and , respectively. The so generated data was taken as an input for both reconstruction methods. In this case the exact value of the over-expression vector *μ *was used as an extra input parameter for the NIR reconstruction. In reality this exact value remains unknown. Results were produced for 20 statistically identical realizations of networks for every connectivity ⟨⟩ ∈ {1, 3, 5}. All the networks provided very similar results, only one for every connectivity is shown in Figure 2. Here we compare the results of our reconstruction method with the NIR algorithm for in-silico experiments. The left panel of the figure shows the fraction of correctly reconstructed links, for every link type (F_+_, F_- _and F_0_) as well as their sum *F*. The colors blue and green represent the NIR and the proposed method, respectively. The pure-chance threshold is shown to emphasize the significance of the result. The right panel shows the extended Matthews correlation coefficient. For the Matthews correlation coefficient the pure-chance threshold is constant at zero.

### Reconstruction of the E. coli SOS network

**Figure 3:**
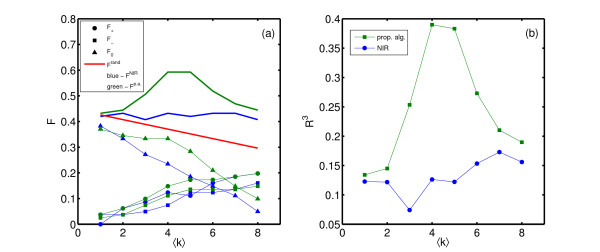
**Network reconstruction and comparison: E. coli**. Reconstruction results for the proposed algorithm (green lines) and NIR (blue lines) for E. coli. The results for the fraction of correctly reconstructed links (a), and the extended Matthews correlation coefficient (b) are shown. In (a) circles denote the fraction of positive links *F*_+_, squares the fraction of negative links *F*_- _and triangles no links *F*_0_. The red line represents the gambling threshold *F*^*rand*^.

**Figure 4:**
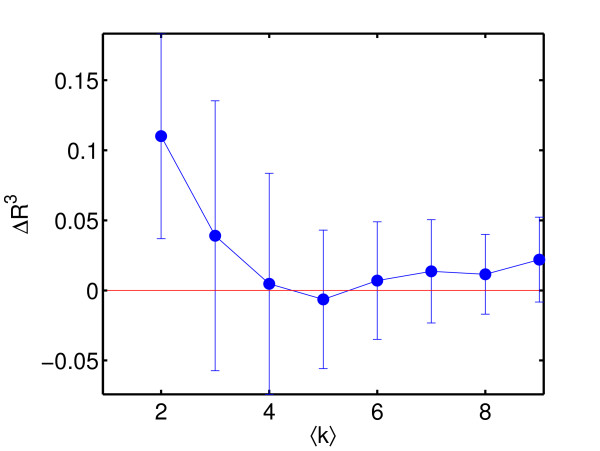
**Matthews correlation coefficients versus average connectivity**. The degree of reliability of how the proposed algorithm outperforms NIR is demonstrated by how much the extended Matthews correlation coefficient *R*^3 ^for the proposed algorithm exceeds the one for NIR. For Δ*R*^3 ^= , ⟨Δ*R*^3^⟩ ± *σ*(*R*^3^) is plotted, where *σ *is the standard deviation of Δ*R*^3^. Mean and standard deviation have been computed from 20 realizations of networks of size *N *= 10 and connectivity ⟨*k*⟩ = 3.

Although our reconstruction method showed better results tested on in-silico networks than NIR, the true value of any reconstruction potential can be shown just on the real biological data. When testing both methods on *E. coli *data, as shown in Figure 3, our reconstruction method outperforms NIR more visibly, in both performance measures. To stress the difference in the quality of reconstruction we present p-values of given correlation coefficients between the real and reconstructed networks. Given the sample size *K *= 81, i.e. the number of links to be reconstructed, and a ⟨*k*⟩ = 4 (known experimental value), the p-value of correlation coefficient  = 0.14 for NIR is *p*_*NIR *_= 0.2126, while the p-value of correlation coefficient *R*^3 ^= 0.4 for our method is p = 0.002. For *R*^3 ^values see Figure 3, at ⟨*k*⟩ = 4. Our reconstruction leads to a network which significantly correlates better with the experimentally known biological network. This is demonstrated in Figure 4 where mean and standard deviation of *δR*^3 ^=  is plotted for 20 realizations of networks of size *N *= 10 and connectivity ⟨*k*⟩ = 3.

The computational time needed to perform the NIR algorithm on this particular 9 node network is of order of magnitude of 1 minute, while our approach takes less than a second, both performed on a standard personal computer. The NIR algorithm is unable to cope with reconstruction of significantly larger gene regulatory networks, both from the time or memory consumption, while our method can easily deal with larger network sizes. Because of typically high levels of noise and uncertainty in biological data collected throughout actual experiments, the robustness of a method is of crucial importance. We tested both the NIR and our algorithm in the following way: We took the *E. coli *data and added Gaussian noise (the noise amplitude, i.e. the standard deviation of the noise, was chosen to be 1.5 percent of the average amplitude of the input data). We produced 100 perturbed datasets, reconstructed the network with both, the proposed algorithm and NIR, and counted the number of links that have changed with respect to the network reconstructed from the unperturbed data, i.e. links where either - → +, 0 or + → -, 0 or 0 → ±. While for NIR 32.7 percent of the links changed on average for the proposed algorithm only 21.3 percent of the links were classified differently. In absolute numbers: for NIR 26.59 links and for the proposed algorithm 17.25 links were classified differently.

## Discussion

From the right panel of Figure 2, showing the Mathews correlation coefficient for the in-silico experiments, it can clearly be seen that for the fraction of correctly reconstructed links our method performs about equally well than NIR for very sparse networks (⟨*k*⟩^*model *^= 1) and outperforms it for more densely connected networks. Looking at the fractions of correctly reconstructed links, one notices a slightly better performance of our algorithm, while for the extended Matthews correlation coefficient the difference is much more notable. To understand this difference, one has to take a closer look at the type I and type II errors of both methods. While the NIR algorithm makes almost the same number of reconstruction errors of all types, there is a clear distinction in errors made by our reconstruction algorithm. The vast majority of errors are made by assuming that there is a link (positive or negative) between two genes, while in the real case there is none, and vice versa. Only a few mistakes are made where the real positive link is reconstructed as negative, or vice versa. This is an additional asset of the proposed reconstruction algorithm.

In Figure 3 one can easily notice that both reconstruction methods, the proposed one and NIR, applied to in-silico data have their maxima in performance when the input average degree equals to the true one, ⟨*k*⟩ = ⟨*k*⟩^*model*^, which can be seen as an additional consistency check of the algorithm. On the other hand, after applying both reconstruction methods on *E. coli *data, just the proposed reconstruction algorithm shows its performance maximum at the ⟨*k*⟩ = ⟨*k*⟩^*E*.*coli *^point, while the NIR method shows similarities in behavior to the pure-chance reconstruction.

**Figure 5:**
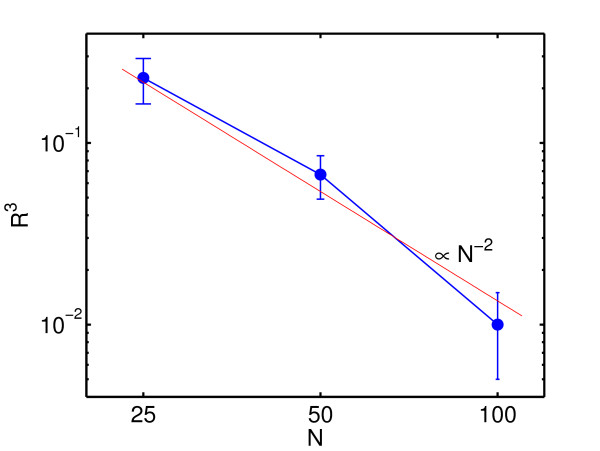
**Correlation coefficients versus network size**. The averages and standard deviation of the associated *R*^3 ^correlation coefficients have been computed and are plotted double logarithmically versus *N*. The straight line is the function *AN*^-2 ^with *A *= 135 and demonstrates that a decay of *R*^3 ^can be explained by power -2 of the network size, which agrees well with the fact that () links have to be estimated from maximal *N *independent experiments. Networks of size *N *= 25 with average connectivity ⟨*k*⟩ = 5, *N *= 50 with ⟨*k*⟩ = 10, and *N *= 100 with ⟨*k*⟩ = 30 have been sampled and reconstructed for the assumed values of *k *= 1,..., *N*.

**Figure 6:**
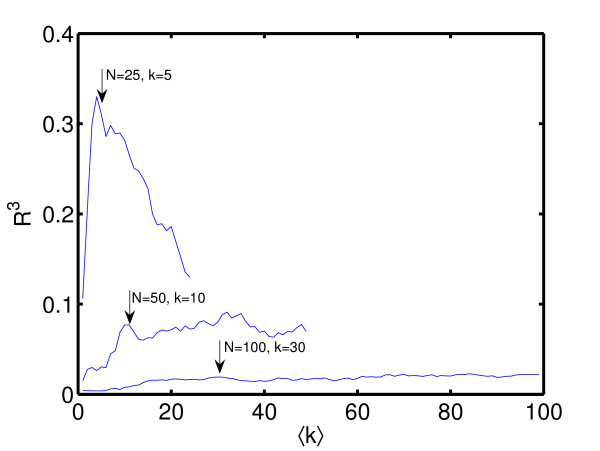
**Size dependence of correlations versus average connectivity**. As in Figure 6 networks of size *N *= 25 and an average connectivity ⟨*k*⟩ = 5, *N *= 50 with ⟨*k*⟩ = 10 and *N *= 100 with ⟨*k*⟩ = 30 have been sampled and reconstructed for the assumed values of *k *= 1,..., *N*. For *N *= 25 *R*^3 ^still shows a pronounced peak around the true value ⟨*k*⟩ = 5 (only missed by one). For *N *= 50 this peak is almost washed out though there is still a residual peak at the correct position. *N *~50 therefore poses a realistic upper limit for the proposed algorithm to reliably outperform pure gambling.

Although the proposed algorithm is fast and can in principle handle very large networks it is of course unrealistic to assume that the algorithm in its present form can reconstruct networks of realistic genome sizes any better than pure chance. This is due to the fact that basically () link weights have to be estimated. Theoretically *N *independent over-expression experiments could in principle suffice to provide a sufficient number of equations to solve the purely linear problem exactly. However, contributions of noise and deviations of the real dynamics from a purely linear dynamics will enter the equations proportionally to the degrees of freedom and corrupt the solution. Any measure that adequately reflects the number of correctly reconstructed links, e.g. *R*^3^, therefore should not decay faster with the network size than with a power of *N*^-2^. In Figure 5 and 6 we demonstrate this fact for the proposed algorithm and the *R*^3 ^correlation coefficient. Figure 6 also demonstrates that while the maximum of *R*^3 ^is still pronounced for *N *= 25 and only misses the correct value of ⟨*k*⟩ by Δ*k *= 1, the pronounced maximum of *R*^3 ^around the true value of ⟨*k*⟩ is almost lost for *N *= 50. *N *= 50 therefore represents a rough estimate of the upper limit of the network size for the proposed algorithm performs better than pure gambling.

## Conclusion

We introduced a reverse engineering procedure for gene regulatory networks, applicable on an experimental setup where all the genes belonging to a genetic (sub)network are being over-expressed one after the other, after which gene-chip measurements in the steady state are taken. We showed the reconstruction performance on both *in-silico *and biological data. The method is applicable to large networks, both from the computational memory or computational time point of view, which might be a problem for algorithms limited by combinatorial explosions. However the increasing lack of independent experimental information with growing network size practically limits networks to sizes *N *≤ 50. However, due to the superior time characteristics, large networks could in principle be decomposed into overlapping subnetworks. These subnetworks can be inferred by the proposed algorithm and then merged together in an adequately chosen post processing step.

Except from technical benefits, the philosophy of our reconstruction method complies perfectly with the biological goals of conducting over-expression experiments. In contrary to the NIR algorithm or similar reconstruction methods, where the final solution is a network, where every link has the same significance, our method ranks the reconstructed links by their influence, which might be a very important issue in experimental gene interaction-detection Instead of randomly picking the links out of a given reconstructed topology, here one can select interaction-links with the highest weights. This again ameliorates the consequences of not knowing the real network connectivity ⟨⟩ a priori. While selecting a good value for ⟨*k*⟩ is crucial for getting reliable networks, it will not influence the ordering of the links by importance in the proposed algorithm. In other words, no matter which ⟨*k*⟩ is taken, the set of ranking of reliable links will not change.

Another shortcoming of the NIR algorithm is the fact that the resulting network has a trivial, unrealistic degree distribution, a delta function, *δ*(*k *- k*). Thus, detecting genetic hubs, peripheral genes, or any other topologically important genes in the network is practically impossible. The proposed method does not a priori restrict the topology of the reconstructed network except for the average degree ⟨*k*⟩ which is important for the thresholding only.

For successful reconstruction the NIR algorithm needs as an external input information on external perturbation, which is in most realistic cases at best only approximately known. In our in-silico experiments we have provided the exact information for NIR; even then the NIR algorithm was outperformed.

## Authors' contributions

The method was mainly developed by DH based on theoretical foundations and ideas provided by RH and ST. All numerical was done by DS The paper was written mainly by ST and RH. All authors read and approved the final manuscript.
